# Editorial: Reviews and advances in cutting edge microscopy and imaging techniques in membrane trafficking and organellar dynamics

**DOI:** 10.3389/fcell.2024.1493620

**Published:** 2024-10-07

**Authors:** Veronika Huntosova, Marco Andreana, Alexandre A. Mironov, Duarte C. Barral

**Affiliations:** ^1^ Center for Interdisciplinary Biosciences, Technology and Innovation Park, Pavol Jozef Šafárik University in Košice, Košice, Slovakia; ^2^ Center for Medical Physics and Biomedical Engineering, Medical University of Vienna, Vienna, Austria; ^3^ IFOM ETS—The AIRC Institute of Molecular Oncology, Milan, Italy; ^4^ iNOVA4Health, Faculdade de Ciências Médicas, NMS, FCM, NOVA Medical School, Universidade NOVA de Lisboa, Lisboa, Portugal

**Keywords:** membrane trafficking, microscopy, organelle dynamics, super-resolution microscopies, electron microscopy

Membrane trafficking pathways play key roles in essential cellular processes including signal transduction, organelle transport, protein sorting and membrane remodeling, as well as cell division, differentiation, motility, and cell death. Several limitations must be overcome to reliably analyze and quantify these processes, and advanced microscopic techniques and approaches have been developed. Current imaging techniques for studying membrane trafficking face several significant challenges, particularly in terms of spatial and temporal resolution, phototoxicity, and the ability to track multiple components simultaneously. Membrane trafficking events such as vesicle budding, transport, and fusion, often occur at nanoscales and at very fast timescales, which conventional light microscopy cannot fully resolve due to the diffraction limit and slow acquisition speeds. This makes it difficult to visualize the small vesicles and dynamic processes involved. Another challenge is phototoxicity and photobleaching, as the constant exposure to light can damage living cells and reduce signal intensity over time, limiting the duration for which dynamic processes can be observed. Imaging at greater depths in tissues also poses a problem, as light scattering and absorption reduce both image clarity and resolution. Additionally, many trafficking processes involve multiple proteins, lipids, and other cargo that must be tracked simultaneously, but current fluorophore technology struggles with spectral overlap, limiting the ability to differentiate multiple components in real time. To overcome these obstacles, a number of advanced techniques have emerged. Super-resolution microscopy methods such as stochastic optical reconstruction microscopy (STORM) and photoactivated localization microscopy (PALM) offer significantly improved spatial resolution, breaking the diffraction limit and enabling visualization of smaller structures. Light-sheet microscopy and adaptive optics improve temporal resolution by allowing faster imaging of live samples with lower phototoxicity. Phototoxicity and photobleaching are further reduced by using near-infrared and two-photon microscopy, combined with more stable fluorophores. Additionally, multiplexing strategies such as spectral unmixing and fluorescence lifetime imaging microscopy (FLIM) enable the tracking of multiple components by distinguishing fluorophores either by their emission spectra or fluorescence lifetimes. These advances offer promising solutions to the current limitations, allowing for more precise, detailed, and functional insights into membrane trafficking dynamics. However, there are still limitations in the microscopic analysis of dynamic events in living cells, namely, regarding spatial and temporal resolution, and the capacity to extract relevant information for the understanding of pathophysiological processes.

Here, we aim to highlight recent progress in this area, while emphasizing important directions and new possibilities for future research. Cutting edge microscopic techniques have been applied to study cell migration, intercellular communication and visualization of membrane proteins, as well as organelle biology at super-resolution, leading to a better understanding of their role in homeostasis and pathology.

The current Research Topic in Frontiers in Cell and Developmental Biology includes four articles from 19 authors, covering cutting-edge imaging methodologies of membrane transport and organelle dynamics with promising applications in a wide range of biological and medical fields. A total of three original research and one methods article in this research area were published. The Research Topic involves different areas of research, as further detailed below.

Cell migration is one of the crucial steps involved in cancer invasion, as well as wound healing. Besides strong efforts, available methods for cell migration analysis often lack high reproducibility or resistance to different stress conditions. Indeed, the most used are performed in a monolayer and, therefore, do not mimic tissue architecture. Joshi et al. have developed 3D-printed biocompatible inserts that enable real-time imaging of cells in 24-well plates. These inserts are suitable for high-throughput cell migration assays under different conditions and allow high reproducibility. Furthermore, they created a macro script on the ImageJ platform that enables rapid automated analyses of thousands of images. The combination of these approaches provides a key technological advance in cell migration studies with high simplicity, reproducibility and robustness.

The plasma membrane is essential to regulate the composition of the intracellular environment, and for intercellular communication. This important cell structure ensures the uptake of nutrients and ligands through membrane receptors that activate signaling pathways, as well as waste removal. Due to the diffraction limit of light microscopy, it is necessary to use advanced light or electron microscopy (EM) to study the phospholipids that compose the plasma membrane bilayer. However, EM does not allow to investigate the link between membrane proteins and physiological processes due to the nature of the sample processing involved. Puchkov et al. have adapted correlative super-resolution light and platinum replica electron microscopy (CLEM-PREM), allowing the detection of plasma membrane proteins and the collection of structural information of the plasma membrane and the cytoskeleton. This technique was combined with super-resolution stimulated emission depletion (STED) fluorescence microscopy and scanning electron microscopy (SEM) to improve navigation and obtain parameters that allow the correlation of images of the same sample. Thus, the proposed approach makes it possible to study plasma membrane-related processes and can extract quantitative information based on appropriate analytical techniques. Indeed, it has great potential to identify molecular details involved in membrane remodeling of organelles like mitochondria, endoplasmic reticulum and lysosomes, as well as cytoskeleton remodeling.

Mitochondria are organelles with unique ultrastructural properties formed by double membranes and characteristic cristae, in which protein complexes of the respiratory chain are located. These compartments control oxidative phosphorylation and the level of oxidative stress in cells, and their morphology can change under specific conditions. Loriette et al. used enhanced speed structured illumination microscopy (Fast-SIM) to investigate mitochondrial dynamics in Chlorin-e6 mediated photo-oxidation. Such an approach yields qualitative and quantitative information about mitochondrial membranes, as well as mitochondrial length, mean square displacement, fission, and fusion. This opens up new possibilities for understanding and investigating the etiology of mitochondrial disorders and, due to Fast-SIM versatility, other organelles can also be studied.

Optical imaging of small structures in living cells is challenging due to the visible light diffraction limit. However, precisely detected optical images contain complex and complete information about imaged objects. Miyashiro et al. developed a methodology for reconstructing the structure of an object from optical microscopy images. In this work, they used single-photon counting, complete noise elimination, and a likelihood-based restoration algorithm to achieve exceptionally high spatiotemporal resolution by super-resolution confocal live imaging microscopy (SCLIM). They present 4D images obtained by capturing sub-diffraction limit structures of organelle and vesicle dynamics in living cells, at the millisecond level. The data obtained during the detection includes precise mathematical information about probability, which can be further used for sophisticated analysis.

In summary, this Research Topic unveils novel imaging methodologies that can be used to study cellular processes including cell migration, membrane and cytoskeleton remodeling, and organelle dynamics. These new methodologies result from improvements at the level of light microscopy, EM or a combination of both, and enhance our capacity to study the mechanisms involved in dynamic cell events. There is little doubt that further improvements in cell imaging yet to come will allow us to keep shedding light on the fascinating complexity of membrane trafficking and organelle dynamics.

